# Neural Network-Oriented Big Data Model for Yoga Movement Recognition

**DOI:** 10.1155/2021/4334024

**Published:** 2021-10-30

**Authors:** Hui Wang

**Affiliations:** School of Physical Education, Qingdao University, Qingdao, Shandong, China

## Abstract

The use of computer vision for target detection and recognition has been an interesting and challenging area of research for the past three decades. Professional athletes and sports enthusiasts in general can be trained with appropriate systems for corrective training and assistive training. Such a need has motivated researchers to combine artificial intelligence with the field of sports to conduct research. In this paper, we propose a Mask Region-Convolutional Neural Network (MR-CNN)- based method for yoga movement recognition based on the image task of yoga movement recognition. The improved MR-CNN model is based on the framework and structure of the region-convolutional network, which proposes a certain number of candidate regions for the image by feature extraction and classifies them, then outputs these regions as detected bounding boxes, and does mask prediction for the candidate regions using segmentation branches. The improved MR-CNN model uses an improved deep residual network as the backbone network for feature extraction, bilinear interpolation of the extracted candidate regions using Region of Interest (RoI) Align, followed by target classification and detection, and segmentation of the image using the segmentation branch. The model improves the convolution part in the segmentation branch by replacing the original standard convolution with a depth-separable convolution to improve the network efficiency. Experimentally constructed polygon-labeled datasets are simulated using the algorithm. The deepening of the network and the use of depth-separable network improve the accuracy of detection while maintaining the reliability of the network and validate the effectiveness of the improved MR-CNN.

## 1. Introduction

Gigabytes of images are generated every day in the Internet, which contain a huge amount of information. People need to process these images in order to browse and retrieve them effectively. Image retrieval has become very active in related research areas since the 1970s. The advancement of image retrieval is also inseparable from the development of database management systems and the effective promotion of computer vision as a field. Most of the information received by humans comes from vision, and vision-based image retrieval is particularly important. Among the components of image retrieval, human action behavior classification is a very important part, and an in-depth investigation into human action image classification can improve the efficiency of retrieval containing human action images [1–4]. Research related to image-based human action recognition in the field of pattern recognition has become a cutting-edge research topic. Human action recognition focuses on recognizing the action or behavior of a person from a single image. Given the limited sources of information and the complex background of images collected from the web and the presence of a large number of still images on the web, action recognition based on still images requires the development of robust and effective methods to better understand the web images for image retrieval or search. At the same time action recognition has great utility and broad application prospects [5–8]. Action recognition systems can be subsequently applied to exercise data evaluation, intelligent training assistance, etc., for example, calculating exercise exertion, training scoring, and virtual coach teaching [9–12].

Yoga, as a convenient and fashionable form of fitness, can relieve anxiety and regulate mental state and enhance body immunity [13, 14]. When people learn yoga, the mainstream learning method is still to enroll in offline yoga training courses, but the practice time and venue are not fully free, and the learning content and progress are usually mastered by the teacher, which brings many inconveniences to the workplace people's exercise in [15]. At the same time, the current level of instructors varies, and the professional quality of teachers is more difficult to guarantee when full understanding is not conducted, so many people choose to find resources from the Internet for self-learning. This way is very convenient, but the accuracy and scientific nature of the exercise cannot be guaranteed, and unreasonable exercise habits and wrong postures can cause physical injury, which is contrary to the original purpose of exercising [16–18].

In order to solve the problem, this paper is divided into the following five chapters: Section 1 briefly introduces the research background of this paper, the current status of the research, and the structure of the paper; Section 2 briefly introduces the research progress and shortcomings of yoga movement recognition and also elaborates and describes the significance and main contents of this paper.  Section 3 specifically introduces the MR-CNN-based yoga action recognition network. The feature pyramid technique is used in the feature extraction part to improve the performance of the network in multiscale target detection. Regions of interest are extracted using a region candidate network for target classification and detection after passing RoI Align, while images are segmented using mask branches. Finally, the improved deep backbone network and the improved mask branching network are applied to the yoga action recognition network to improve the accuracy of target detection.  Section 4 firstly introduces the evaluation indexes of the adopted recognition performance and then validates and evaluates the recognition effect of the MR-CNN-based yoga action recognition model. The experimental results show that the scheme proposed in this paper has high accuracy for yoga movement recognition.  Section 5 briefly summarizes the research and practical contents of this paper and describes the shortcomings of the research contents and the outlook of future research work.

## 2. Related Works

Yoga action recognition belongs to a type of action in human action recognition, which is mainly applied in the field of sports and can also be extended to other types of action recognition [19]. Human action recognition is jointly implemented by many different and intersecting disciplines, such as machine learning, artificial intelligence, sensor technology, and computer vision. There are different ways of acquiring human movements, according to which they can be classified as, first, human movement recognition based on wearable sensors and, second, human movement recognition based on vision [20].

Wearable sensors use sensors fixed to key locations on the human body to capture motion data and analyze the computational data to recognize the actions performed by the human body. This type of action recognition system has more comprehensive data analysis capability, but requires higher equipment and expertise, and the acquisition of parameters is more inconvenient. Obviously vision-based action recognition has a higher universal applicability. Meanwhile video and image have become the main carriers of information due to the development of network technology, and the activities of human society are mainly recorded in video and image. Whether from the perspective of entertainment, sports, surveillance, and security, the study of human action recognition in images is of great significance. Human action recognition is mainly used to analyze and understand human actions by processing and analyzing image data or image sequences.

The researcher divided human motion recognition into four stages: initialization system, bone extraction, pose estimation, and pose recognition [21]. The researcher designed a pose recognition model as based on the hidden Markov model; this model models human motion in a cascade form, and the main feature is the use of expectation maximization algorithm in order to ensure reliability and accuracy [22]. Some researchers designed a yoga pose recognition system for self-training and used a star algorithm to extract the human star skeletal point vector to detect the ongoing yoga poses [23]. The researcher designed an electronic yoga teaching system based on somatic devices, using the Hausdorff algorithm and others to assess the similarity of poses and determine the names [24].

A variety of different features are widely used in yoga action recognition, including location, contour, and time-space features, which are specifically classified as static, dynamic, and spatiotemporal features [25–27]. Static features are color, size, contour, edge, object shape, and depth from which behavioral details and contour states are extracted. Dynamic features are direction, velocity, trajectory, etc., from which motion patterns are obtained. Spatiotemporal features are used for video and image sequences to extract 3D data models such as spatial and spatiotemporal cubes.

Although many scholars have achieved certain research results, the accuracy of yoga action recognition still needs to be improved as it is easy to lose the preliminary feature information in the process of action recognition. In order to further improve the accuracy of yoga action recognition, the significance of this paper is to improve the accuracy of yoga action recognition and fully exploit the existing resources to further promote the development of intelligent sports industry. In view of the fact that the research on yoga movement recognition technology is in the development stage, this paper carries out the research on yoga movement recognition technology based on MR-CNN. This paper provides theoretical support for yoga movement recognition, which is of great practical significance to the field of intelligent sports and has long-term application prospects for the development of intelligent software and hardware.

## 3. MR-CNN-Based Yoga Movement Recognition Network

The yoga action recognition task includes recognizing the name of yoga actions, locating the position of human yoga actions in the image, and also segmenting the yoga actions. Action classification and localization belong to two tasks of target detection, and the most representative algorithm in target detection is faster region candidate convolutional network (Faster R–CNN). In order to achieve further yoga action segmentation tasks, this paper extends the Faster R–CNN and selects the MR-CNN algorithm as the detection and segmentation network for the study. Considering that MR-CNN achieves action classification and localization with the addition of mask branch for segmentation of yoga actions, this chapter uses MR-CNN to implement the detection and segmentation task, improves the feature extraction backbone network, uses the improved depth residual network with the addition of batch normalization layer instead of the traditional depth residual network, improves the mask branch, and uses the depth-separable convolution instead of the traditional convolution. Compared with the traditional convolutional neural network, the residual network adds shortcut connections and adds a batch normalization layer to the residual block to improve it. The superimposition of the residual block constitutes an improved deep residual network. The improved deep residual network can achieve a smaller average error after multiple trainings and has a higher detection accuracy.

### 3.1. Faster Region Candidate Convolutional Neural Networks

Currently, target detection can be classified into two types of neural network algorithms based on regression and candidate region. The neural network algorithms based on regression have a high computational efficiency and perform rule and dense sampling using features such as aspect ratio, scale, and position to detect targets. However, the detection accuracy is lower compared to the neural network algorithms based on candidate region. One of the main reasons is due to the category imbalance problem. This paper focuses on a target detection algorithm based on a two-stage detector. In the first stage of the two-stage approach is the generation of candidate target frames, and in the second stage further classification and regression are performed. The optimal performance is obtained for the two-stage approach on several challenging datasets such as PASCAL VOC and MS COCO. The network framework for faster region candidate convolutional neural contains two parts, the extracted candidate frame part and the target detection part. It can be divided into four parts: first, using the image classification model as the backbone network to extract the image features; second, inputting the image features to the region candidate network to obtain the candidate regions; third, inputting the results obtained from the previous two steps, i.e., image features and candidate regions, to the RoI Pooling layer to obtain the integrated candidate region features; fourth, predicting the bounding box of the object and the class of the object based on the candidate region features.

Region proposal network (RPN) uses a convolutional network to construct candidate regions without considering categories by sliding convolution over the features. The network uses classifiers with only two categories: the category with targets and the category without targets. RPN performs *k*a RoI prediction for each point in the feature map. Therefore, RPN outputs 4 × *k*one RoI coordinate and 2 × *k*one target score for each pixel location.

**Figure 1:**
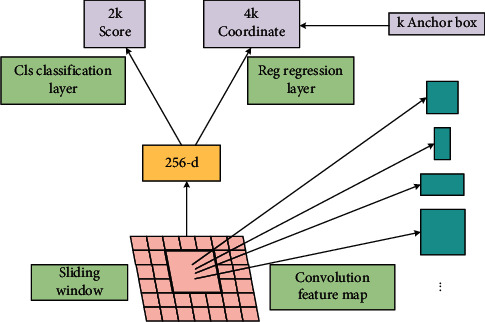
Regional candidate network framework.

A set of anchor points is generated for each point on the processed convolutional feature map, and the anchor points generated on the feature map also need to be mapped to the size of the input image in the end. The feature extraction process includes only convolution and pooling layers, so the final dimension of the feature map is proportional to the original image. If the size of the image is*w* × *h*, then the final feature map is compressed to a size of *w*/*r*and *h*/*r*, where *r*is the subsampling rate. If an anchor point is defined at each spatial location on the feature map, the anchor points of the final image will be separated by *r*one pixel, and Figure 1 shows the concrete framework of the network implementation.

Network specific implementations are as follows:

1. Firstly, the features of the original image are extracted using a convolutional neural network, which is a commonly used image classification backbone.
2. Formation of feature maps is as follows:
3. In the feature map generated by the sliding scan, the sliding window size is *n* × *n*, and the lower dimensional feature vector obtained in the next step is derived from the convolutional layer mapping the sliding window position. The sliding window is designed as3 × 3. Although *n*=3 looks small, each rectangular window frame is perceptible to a large extent considering the small size of the higher-level feature map itself. After mapping to the low-dimensional feature vector using Rectified Linear Unit (ReLU), and considering the *k*kinds of possible anchor frames for each sliding window position without considering beyond the bounding anchor frame, nine candidate regions will be predicted with feature map size *W* × *H*, and the region proposal will yield *W* × *H* × *k*one.
4. After the region suggestion, there are two fully connected layers, regression layer and classification layer, in which the input is a low-dimensional feature vector, and the regression layer serves to generate the bounding box corresponding to*k*, *k*, (*x*, *y*, *w*, *h*), to ensure that the candidate box does not exceed the image boundary, to crop the part that exceeds it to be close to the edge and to determine whether the candidate region is the background part or the foreground part and score it. In order to accurately represent the coordinates of the candidate regions, the number of results in the window regression layer is 4*k*, and the number of results in the classification layer is 2*k*, indicating the probability of the candidate *k*regions being background and foreground, respectively.

The goal of the task is to unify the bounding box P and the ground truth G. The idea is not to learn the specific coordinates of *G*, but to learn the scale of the deformation performed during the transformation of the bounding box. The idea of the transformation is to move the coordinates of the bounding box positioning (*x*, *y*) and to scale the size of the bounding box according to the ratio (*w*, *h*).

Suppose the original predicted bounding box is *P*(*x*, *y*, *w*, *h*), and the calibrated bounding box is*G*(*x*, *y*, *w*, *h*), where *x*, *y*, *w*, *h* denote the coordinates of the center point of the bounding box and the width and height, respectively. In order that the regression window obtained after the mapping transformation of the bounding boxPcan be closer to the real window *G*, learning the transformation relation of the bounding box is the main goal. That is, given (*P*_*x*_, *P*_*y*_, *P*_*w*_, *P*_*h*_), find a mapping *f*, such that

(1) $$\begin{array}{c} \left(P_{x},P_{y},P_{w},P_{h}\right)=\left(\hat{G_{x}},\hat{G_{y}},\hat{G_{w}},\hat{G_{h}}\right)\approx \left(G_{x},G_{y},G_{w},G_{h}\right). \end{array}$$

Perform the translation first:

(2) $$\begin{array}{c} \left(\Delta x,\Delta y\right),\Delta x=P_{w}d_{x}\left(P\right),\Delta y=P_{h}d_{y}\left(P\right), \end{array}$$

(3) $$\begin{array}{c} \hat{G_{x}}=P_{w}d_{x}\left(P\right)+P_{x}, \end{array}$$

(4) $$\begin{array}{c} \hat{G_{y}}=P_{h}d_{y}\left(P\right)+P_{y}. \end{array}$$

Then, perform scaling:

(5) $$\begin{array}{c} \left(S_{w},S_{h}\right),S_{w}=P_{w}d_{w}\left(P\right),S_{h}=P_{h}d_{h}\left(P\right), \end{array}$$

(6) $$\begin{array}{c} \hat{G_{w}}\;=P_{w}e^{d_{w}\left(P\right)}\hat{G_{h}}\;=P_{h}e^{d_{h}\left(P\right)}. \end{array}$$

Therefore, it can be seen from the above formula that the four transformation parameters to be learned are *d*_*x*_(*P*), *d*_*y*_(*P*), *d*_*w*_(*P*),  and *d*_*h*_(*P*). P is not the true value *G* but the predicted $\hat{G}\;$ value after the transformation of the four parameters; then, the objective function can be expressed as *d*_*∗*_(*P*)=*w*_*∗*_^*T*^Φ_5_(*P*), Φ_5_(*P*) is the feature vector of the input Proposal,*w*_*∗*_^*T*^ is the parameter to be learned, where *∗* denotes*x*, *y*, *w*, *h* , and*d*_*∗*_(*P*) is the calculated predicted value. According to the distance relationship between the predicted value and the true value, the loss function is obtained as

(7) $$\begin{array}{c} \text{loss}=\sum_{i}^{N}{\left(t_{*}^{i}-{\hat{w}}_{*}^{T}\Phi_{5}\left(P^{i}\right)\right)}^{2}. \end{array}$$

The function optimization objective is

(8) $$\begin{array}{c} w_{*}=\arg\min\sum_{i}^{N}{\left(t_{*}^{i}-{\hat{w}}_{*}^{T}\Phi_{5}\left(P^{i}\right)\right)}^{2}+\lambda {\left\|\hat{w_{*}}\right\|}^{2}. \end{array}$$

**Figure 2:**
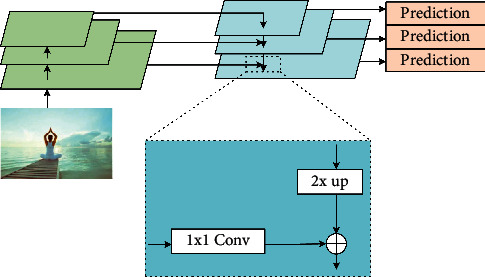
Feature pyramid network structure.

In the research of action recognition, the depth and step length of the network usually restrict each other. Common solutions to this problem include image pyramids and feature layering. The multiscale training and testing of image pyramids are time consuming and computationally intensive, making it difficult to apply in practice. Feature layering, i.e., each layer predicts the detection results for the corresponding scan resolution separately, allows different feature layers to learn the same semantic information. However, since in convolutional neural networks different layers correspond to different semantic features at their respective levels, shallow networks with high resolution learn more detailed features, and deeper networks with low resolution learn more semantic features. The feature pyramid network (FPN) improves on this problem by introducing feature maps for each resolution into the latter one scaled by twice the resolution to do the summation operation. Since this method only adds additional cross-layer connections to the original network, it adds almost no additional time and computation in practical applications. The network structure is characterized by the ability to fuse the features of each layer and strengthen the semantic information while enhancing the spatial information, and the network structure is shown in Figure 2.

The left-hand model of the FPN structure is the bottom-up part, with bottom-up paths for feature extraction, using a skeleton network for computation. The model on the right is the top-down part, using nearest-neighbor upsampling for upsampling starting from the highest layer instead of the deconvolution operation, which is simpler to implement and can effectively reduce the training parameters. The horizontal arrow is a lateral connection that fuses the result obtained by upsampling with the feature map generated from the bottom-up.

### 3.2. MR-CNN Framework and Structure

**Figure 3:**
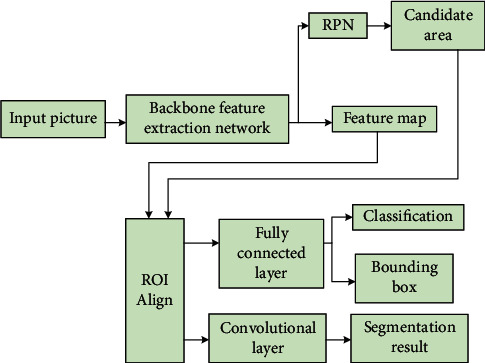
Example partitioning framework structure.

The framework in this paper is extended from the fast region-convolutional network by adding a semantic segmentation branch to perform predictive segmentation for each candidate region, while being parallel to the existing backbone used for classification and bounding box regression, and the overall instance segmentation framework is structured as in Figure 3 where the segmentation task is mainly implemented by the extended branch, which is a full convolutional network acting on each candidate region to perform prediction at the pixel level segmentation regions. While common instance segmentation systems perform classification on top of segmentation completion, MR-CNN is implemented in parallel with classification and segmentation.

RoI pooling is not aligned pixel by pixel, which has little effect on the bounding box but has a significant impact on the accuracy of the mask.

After using the RPN network candidate window, the predicted targets are processed afterwards. Since the predicted regions vary in size and resolution, a uniform quantization operation is required before the extracted features are input to the fully connected layer. Since the network has an FPN part as well as a segmentation image task, the traditional ROI Pooling layer is not suitable, so MR-CNN uses the ROI Align layer for its optimization. The specific steps of traditional ROI Pooling are as follows:

1. Based on the input image, the ROI is mapped to the corresponding position of the feature map, and a rounding operation is performed during the calculation, i.e., the first quantization.
2. The mapped region is divided into parts of the same size, the number of parts divided is the same as the dimensionality of the output, and the forensic operation is performed when the size of each part of the region is calculated, i.e., the second quantization.
3. Maximum pooling operation is performed for each part.

After the above steps, which mainly process the boxes with different sizes, the resultant output feature map is of fixed size, which can achieve ROI, and convolution feature map size does not affect the output feature map size and can improve the processing speed. However, it can be seen that after two quantizations to round the floating-point numbers, the candidate regions originally mapped on the feature map will produce deviations, which will have an impact on the regression localization in the later layers. In order to solve the error caused by the quantization operation, the optimized candidate window processing eliminates the quantization operation and uses bilinear interpolation when obtaining the values on the pixel points of the floating-point coordinates as follows:

1. Iterate over all candidate regions to ensure that the floating-point boundaries are not quantized.
2. Partition the candidate region into*k* × *k* cells, with the same cell boundaries unquantified.
3. For the four coordinate points of the computation area of each partial cell, by bilinear interpolation, perform the maximum pooling operation.

In the target segmentation task, a *L*_*mask*_component is added to calculate the cross-entropy of the predicted target segmented image compared to the conventional detection network. Since the overall network is in multitask learning mode, the loss function is as follows:

(9) $$\begin{array}{c} L=L_{\text{class }}+L_{b\text{ box }}+L_{\text{mask }}, \end{array}$$

where *L*_class _ represents the classification loss, *L*_*bbox*_ is the target detection regression loss, and *L*_mask _ is the segmentation loss.

#### 3.2.1. Classified Losses

During the training process, the target detection network generates an *m*region recommendation window. Let*p* be the probability of correct classification, and the network is used to achieve the multiclassification task, so the cross-entropy function is usually chosen as the loss function, and the formula is as follows:

(10) $$\begin{array}{c} L_{\text{class }}=\sum_{i}^{m}p_{i}\log\;p_{i}+\left(1-p_{i}\right)\log\left(1-p_{i}\right). \end{array}$$

#### 3.2.2. Target Detection Coordinate Regression Loss

For the prediction of edges, which is a regression problem, a squared loss function, or L2 loss, can usually be chosen. The L2 loss function has a high penalty at large errors. Therefore, a slightly more moderate absolute loss function (L1 loss) is used, which grows linearly with error rather than squared. However, this function does not have a derivative at the 0 point, so it may affect the sword collection. A common solution is to segment the function, using a squared function near the 0 point to make it smoother. This segmentation function is called the smooth L1 loss function, or SmoothL1Loss.

#### 3.2.3. Segmentation of Losses

After the target detection network passes through the RPN network and obtains *m* regional recommendation windows, the target branch will output *m*a × a matrices, and the matrix elements are the probability values of [0, 1]. The logarithmic loss function is applied to measure the target segmentation results. The single-pixel point loss is as follows.

The candidate window segmentation image matrix has a dimension a × a, and the overall loss is as follows:

(11) $$\begin{array}{c} L\left(y,p\left(y|x\right)\right)=-y\;\ln\;p\left(y|x\right)-\left(1-y\right)\ln\left(1-p\left(y|x\right)\right). \end{array}$$

a × a is the image matrix dimension, and the overall loss is as follows:

(12) $$\begin{array}{c} L_{mask\;}=\frac{1}{m}\sum_{i=1}^{m}\sum_{j=1}^{a\times a}L\left(y_{i},p\left(y_{i},x_{i}\right)\right). \end{array}$$

To further simplify the network parameters and improve the segmentation accuracy, a separable convolutional neural network is used to replace the normal convolutional structure in the MR-CNN algorithm for mask separation. While the conventional convolution considers the region first and then the channel, the deep separable convolution considers both the channel and the region, which also reduces the required parameters of the network.

A separable convolution is composed of a depth convolution and a point-by-point convolution compared to a conventional convolution. A deep convolution is a set of two-dimensional convolution kernels that perform spatial convolution on each input channel. One channel is responsible for one convolution kernel, so a deep convolution operation requires fewer parameters. Deep convolution learns each channel instead of one convolution kernel corresponding to all channels, allowing for a richer feature set. Point-by-point convolution uses 1 × 1 convolution window operation on the feature map obtained in the previous step to map the output to a specified number of channels.

The size of the convolution kernel of the standard convolution is set as *D*_*k*_ × *D*_*k*_ × *M* × *N*, the input feature map is set as*F*_*in*_ × *F*_*in*_ × *M* , and the output is set as*F*_out _ × *F*_out _ × *N*, where *F*_*in* _ and *F*_*out* _ represent the input and output feature map size, respectively, and *M*, *N* the number of input and output channels, respectively, and the size *D*_*k*_of the convolution kernel. The required computation size for the standard convolution kernel is

(13) $$\begin{array}{c} D_{k}\times D_{k}\times M\times N\times F_{in}\times F_{in\;}. \end{array}$$

Using the depth convolution combined with 1 × 1 the convolution instead of the traditional convolution, the computation of a single depth convolution is

(14) $$\begin{array}{c} D_{k}\times D_{k}\times M\times F_{in}\times F_{in\;}. \end{array}$$

From the above two computational quantities, it can be seen that a single depth convolution is very efficient and less computationally intensive, and it needs to be followed by a 1 × 1 convolution for the linear combination of the output channels. The computational effort of the depth-separable convolution is

(15) $$\begin{array}{c} D_{k}\times D_{k}\times M\times F_{in}\times F_{in}+M\times N\times F_{in}\times F_{in\;}. \end{array}$$

The ratio of the computational effort of the deeply separable convolution to the conventional convolution for equal number of channels and convolution kernels is

(16) $$\begin{array}{c} \frac{D_{k}\times D_{k}\times M\times F_{in\;}\times F_{in\;}+M\times N\times F_{in\;}\times F_{in\;}}{D_{k}\times D_{k}\times M\times N\times F_{in\;}\times F_{in\;}}=\frac{1}{N}+\frac{1}{D_{k}\times D_{k}}. \end{array}$$

Deeply separable convolution parameters are fewer and less computationally intensive, which improves the network performance. Therefore, it is combined with the mask branch in MR-CNN.

## 4. Experimental Results and Analysis

**Figure 4:**
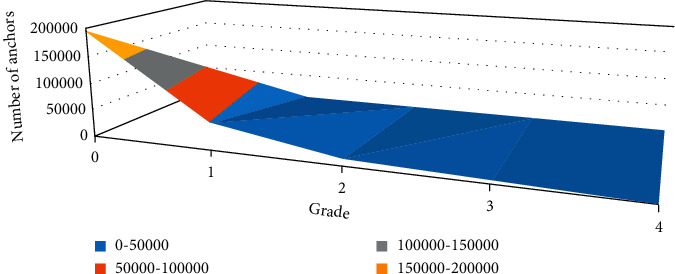
Number of anchor points by level and size of the feature map.

The images of yoga pose in the dataset were sourced from the web and downloaded from the web using a search engine. Due to the lack of a relevant dataset of yoga poses, the downloaded images were manually annotated. The images also contain poses captured from different camera views. Each category of the dataset has an average of 100 images. The images include not only yoga poses with clean backgrounds, but also yoga poses with different backgrounds such as forest, grass, and indoor. The dataset contains 200 images, and 80% of the total number of images are used as the training set and 20% as the test set. The order of the anchors is important in the construction of the dataset, and it is necessary to ensure that the same order is used in the training and prediction phases and to match the order of convolutional execution. For the FPN network, the anchors had to be sorted to make it easy to match the anchors with the output of the convolutional layer that predicts the anchor scores and displacements. First, they are sorted by pyramid level, all anchors in the first level, followed by all second levels, and so on. This makes it easier to separate anchor points by level. Within each level, the anchors are sorted in the order of feature map processing. Typically, the convolutional layer processes the feature maps starting from the top left and moving right row by row.  Figure 4 shows the number of anchor points and the size of the feature map corresponding to each level.

### 4.1. Network Performance Metrics

The performance of network classification recognition is mainly evaluated using a combination of accuracy, precision, recall, and F1 values. The examples in this paper are classified into two types, i.e., positive and negative, and the calculation formulas are

(17) $$\begin{array}{c} \text{accuracy }=\frac{TP+TN}{TP+FP+TN+FN}, \end{array}$$

(18) $$\begin{array}{c} P=\frac{TP}{TP+FP}, \end{array}$$

(19) $$\begin{array}{c} R=\frac{TP}{TP+FN}, \end{array}$$

(20) $$\begin{array}{c} F1=\frac{2\times P\times R}{P+R}. \end{array}$$

The precision rate is denoted by *p* and the recall rate is denoted by R. TP denotes the number of cases that are actually positive and correctly identified as positive, FP denotes the number of cases that are actually negative but incorrectly identified as positive, and FN denotes the number of cases that are actually positive but incorrectly identified as negative. TN denotes the number of cases that are actually negative and correctly identified as negative.

The evaluation metrics used in the target detection task to evaluate the performance of the network are mainly two indices: Mean Average Precision (MAP) and Mean Intersection over Union (MIoU). MAP determines the accuracy of the network in predicting the class of objects in the box, and MIoU determines whether the detection box predicted by the network overlaps with the manual. The MAP determines how accurately the network predicts the object category in the box, and the MIoU determines whether the detection box predicted by the network overlaps with the manually marked box. The target detection sets a threshold to determine whether the target is correctly predicted, and the threshold affects the accuracy and recall rate. The AP curve is obtained by averaging the APs of different categories within the interval [0, 1], and the larger the MAP value, the more accurate the prediction. Complete overlap is the ideal result of network prediction, when the value of intersection-to-merge ratio is 1.

The calculation formula for the cross-merge ratio is

(21) $$\begin{array}{c} IOU=\frac{c\cap G}{C\cup G}. \end{array}$$

C in the formula that represents the labeled target area, and *G* represents the candidate area for network detection. At this point, the larger the value of IOU, the more accurate the detection frame of the framed target and the better the network performance.

### 4.2. Analysis of Results

**Figure 5:**
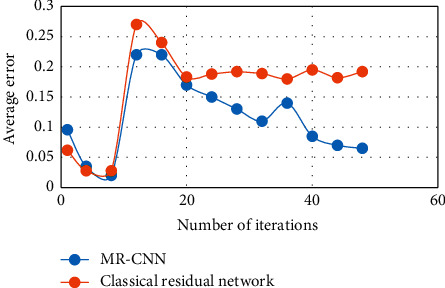
Average error during network training.

Figure 5 shows the comparison of the average error of MR-CNN with the classical residual network during the training process. The figure shows that the average error decreases rapidly during the first few iterations, but the MR-CNN is able to achieve a much smaller average error during the subsequent training process.

**Figure 6:**
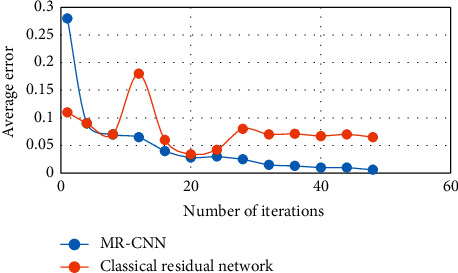
Average error during network testing.

Figure 6 shows the comparison of the average error of the MR-CNN with the classical residual network during the test. The figure shows that after 20 iterations, the improved residual network has a lower average error and performs better during the test.

**Figure 7:**
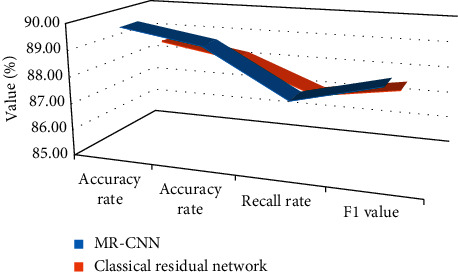
Network identification performance.

The performance of MR-CNN and the traditional deep residual network for yoga action classification is shown in Figure 7. It can be found that the improved residual network has higher recognition performance and better feature extraction performance and can accurately identify the corresponding actions in the images.

**Figure 8:**
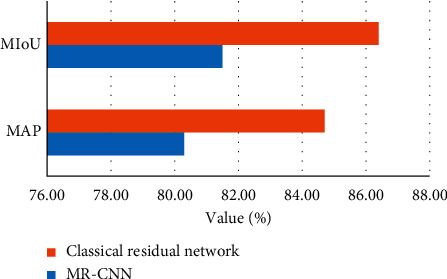
Network detection segmentation accuracy.

The use of a more lightweight depth-separable network in the mask segmentation branch of the improved MR-CNN speeds up the network and improves the accuracy rate. The improved MR-CNN designed in this paper is used to conduct yoga action recognition and detection experiments. During the experiments, test images are fed into the trained network, prediction calculations are performed, and the results of the images output from the network are compared. The respective detection results using MR-CNN and the improved network with the training dataset are shown in Figure 8. The improved network has a stronger learning capability, which can segment the target region more accurately, improve segmentation accuracy, and better fit the predicted segmented edges to the target edges.

**Figure 9:**
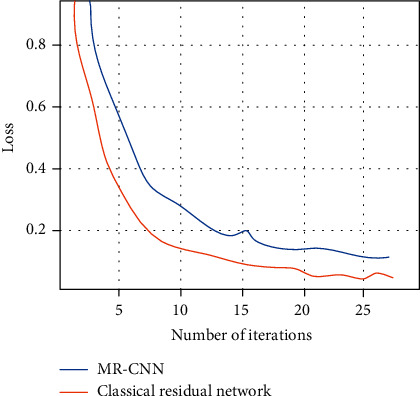
Overall loss function of the network training process.

**Figure 10:**
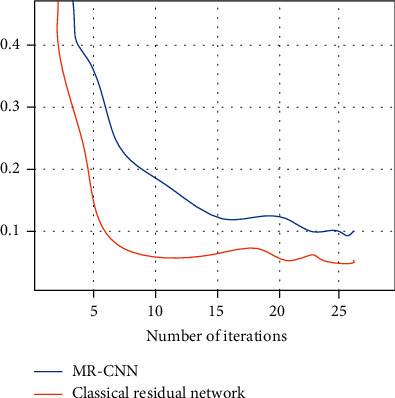
Number of anchor points by level and size of the feature map.

The variation of the loss function during the training process also reflects the performance of the network at the same time. Figures 9 and 10 show the loss profiles of the network during the training process of 30 epoch. It can be seen that the overall loss performance of the improved network is better and the loss function converges faster.

## 5. Summary and Outlook

This paper describes the current situation and problems in the field of image detection and segmentation of yoga poses, and the research designs a target detection and segmentation network. Based on the theoretical basis of convolutional neural network and combined with current related networks, a recognition detection network based on improved depth residual network is proposed, and a network for optimal segmentation of images is proposed in combination with the recognition detection network to realize the yoga action recognition and detection segmentation and candidate region processing network. Experiments show that the proposed scheme in this paper can effectively recognize yoga poses. Some problems have also been discovered during the experiment in this article. For example, the network is slow to decline during the training process, and the network weight parameters may fail to find the global optimal solution. More parameter optimization strategies can be studied in the future to optimize network training. The related field still faces many problems, such that the application of the former MR-CNN mainly focuses on two-dimensional images, and the three-dimensional features of the target can complement the flat image features in complex environments. Follow-up research should explore object detection in combination with 3D technology in order to achieve efficient recognition detection.

## Data Availability

The data used to support the findings of this study are available from the corresponding author upon request.

## References

[1] Juana R. P., CastelánMartínez Osvaldo D., MújicaCalderón Abril J., SánchezRodríguez Martha A., MendozaNúñez Víctor M. (2018). Effect of tai chi on markers of oxidative stress: systematic review and meta-analysis. *International Journal Of Environmental Research and Public Health*.

[2] Solakoglu O., Karatekin B. D., Yumusakhuylu Y., Mesci E., Icagasioglu A. (2019). The effect of yoga asana ‘vrksasana (tree pose)’ on balance in the patients with postmenopausal osteoporosis: a randomized controlled trial ‘vrksasana (tree pose)’ in osteoporosis. *American Journal of Physical Medicine & Rehabilitation*.

[3] Tunuguntla R., Tunuguntla H. S. G. R., Kathuria H., Verma S (2021). Effectiveness of app-based yoga of immortals (YOI) intervention for insomnia in asian population during pandemic restrictions. *International Journal of Environmental Research and Public Health*.

[4] Bryan S., Zipp G., Breitkreuz D. (2021). The effects of mindfulness meditation and gentle yoga on spiritual well-being in cancer survivors: a pilot study. *Alternative Therapies in Health & Medicine*.

[5] Qin X., Ge Y., Feng J. (2017). DTMMN: deep transfer multi-metric network for RGB-D action recognition. *Neurocomputing*.

[6] Mishra S. R., Sanyal G., Sarkar A., Chandra Satapathy S. (2019). Real time human action recognition using triggered frame extraction and a typical CNN heuristic. *Pattern Recognition Letters*.

[7] Alexandros S., Ronald P. (2021). Learn to cycle: time-consistent feature discovery for action recognition. *Pattern Recognition Letters*.

[8] Junjun G. (2018). Basketball action recognition based on FPGA and particle image. *Microprocessors and Microsystems*.

[9] Tracy O. (2021). The experience of trauma center-trauma sensitive yoga training on professional practice of mental health professionals and yoga instructors. *Complementary Therapies in Clinical Practice*.

[10] Lv Z., Xiao F., Wu Z., Liu Z., Wang Y. (2017). Hand gestures recognition from surface electromyogram signal based on self- organizing mapping and radial basis function network. *Biomedical Signal Processing and Control*.

[11] Rao H., Xu S., Hu X., Cheng J., Hu B. (2021). Augmented skeleton based contrastive action learning with momentum LSTM for unsupervised action recognition. *Information Sciences*.

[12] Xu C., Wu X., Li Y., Jin Y., Wang M., Liu Y. (2019). Cross-modality online distillation for multi-view action recognition. *Neurocomputing*.

[13] Amel Ben M., Atri M. (2020). A flexible high-level fusion for an accurate human action recognition system. *Journal of Circuits, Systems, and Computers*.

[14] Ren Z., Zhang Q., Qiao P., Niu M., Gao X., Cheng J. (2018). Joint learning of convolution neural networks for RGB-D-based human action recognition. *Electronics Letters*.

[15] Tsai J. K., Hsu C. C., Wang W. Y., Huang S. K. (2020). Deep learning-based real-time multiple-person action recognition system. *Sensors*.

[16] Wang J., Wang J., Wen X. (2020). A spatio-temporal attention convolution block for action recognition. *Journal of Physics: Conference Series*.

[17] Zhang Y., Sun S., Lei L., Liu H., Xie H. (2021). STAC: spatial-temporal attention on compensation information for activity recognition in FPV[J]. *Sensors*.

[18] Tan T. H., JinHao H., Liu S. H., Huang Y. F., Munkhjargal G. (2017). Using direct acyclic graphs to enhance skeleton-based action recognition with a linear-map convolution neural network. *Sensors*.

[19] Sotoodeh M. S., Hamidreza T. T., Nouchine H., Amandine L. (2020). Preserved action recognition in children with autism spectrum disorders: evidence from an EEG and eye-tracking study. *Psychophysiology*.

[20] Zhong Q. (2019). Robotics - androids; studies from ningbo university of technology further understanding of robotics - androids (research on discriminative skeleton-based action recognition in spatiotemporal fusion and human-robot interaction). *Journal of Robotics & Machine Learning*.

[21] Elharrouss O., Almaadeed N., Al-Maadeed S., Ahmed B., Beghdadi A. (2020). A combined multiple action recognition and summarization for surveillance video sequences. *Applied Intelligence*.

[22] Hajra B. N., Murtaza F., Muhammad H. Y., Sergio A. (2020). Velastin. Multiple batches of motion history images (MB-MHIs) for multi-view human action recognition. *Arabian Journal for Science and Engineering*.

[23] Dong T., Lu Z.-M., Chen X., Ma L.-H. (2017). An attentional spatial temporal graph convolutional network with co-occurrence feature learning for action recognition. *Multimedia Tools and Applications: An International Journal*.

[24] Jospe K., Flöel A., Lavidor M. (2018). The interactive effect of empathy and motor cortex stimulation on hand gesture comprehension. *Neuropsychologia*.

[25] Huang Z. H., Li T., Jiang Y. P., Du L. (2021). Mask R-CNN based style map collar recognition. *Journal of Apparel*.

[26] Kassahun S. K., Kiflie Z., Kim H., Baye A. F. (2019). Process optimization and kinetics analysis for photocatalytic degradation of emerging contaminant using N-doped TiO2-SiO2 nanoparticle: Artificial Neural Network and Surface Response Methodology approach. *Environmental Technology & Innovation*.

[27] Niyas S., Chethana Vaisali S., Iwrin S. (2018). Segmentation of focal cortical dysplasia lesions from magnetic resonance images using 3D convolutional neural networks. *Biomedical Signal Processing and Control*.

